# Botany in Its Relations with Medicine—No. 4

**Published:** 1878-01

**Authors:** W. T. Grant

**Affiliations:** Atlanta, Ga.


### BOTANY IN ITS RELATIONS WITH MEDICINE—No. 4.

By W. T. Grant, M.D., Atlanta, Ga.

It is with a great deal of satisfaction that I present the following very interesting items of information to the profession, which I take from a correspondence prompted by the first article of this series.

Some time since I received a letter from Dr. J. A. Shaffer, of Gainesville, Georgia, enclosing some of the seed of some unknown plant, evidently belonging to the nat. order compositæ. In replying to the letter, I asked him to send me as much of the plant as he could, such as flowers, stem, leaves, etc., so that I might determine what it was. He answered, enclosing some of the inside scales of the involucre, together with some of the cotton-like chaff which grows among the flowers on the receptacle, stating that this was all that he could now send, as he has not recently grown the plant. These parts, with the seed already in my hands, were sufficient to enable me to identify the plant as the cnicus benedictus, but perhaps better known by its common name of blessed thistle.

I now quote from Dr. Shaffer's letter:

"Enclosed you will find a few seed. The history is this: Some years ago I visited an obscure family. They had just moved on a place and in a house that had been vacant for several years, and all around the house had grown up in bushes and weeds. I asked the tenant if he was not afraid to live there, as it had the appearance of being infested with snakes. His reply was that he asked snakes no difference, and that he would nearly as soon be bitten by a snake as scratched by a brier. He then pointed to three dogs that had been bitten by rattlesnakes—one three times, the second twice and the other once—and then spoke of several persons (of whom I had heard as being bitten by rattlesnakes); and the dogs and persons were cured by the use of a few seed, that was a sure cure for snake bites. He then told me that from three to nine seed would cure any snake bite. I procured some of the seed and planted them the next year.

"I have not had the opportunity to use the seed but in one case. Last August I saw a young lady one and a half hours after she had been bitten by a large rattlesnake's pilot (upland copperhead.) The reptile had bitten her twice on the instep. One gill of whisky was at once given. When I saw her I was slow to do anything, and wanted to know what the whisky would do. But as the patient grew worse, I mashed up three seed (she then had double vision) and gave them, which did not appear to exercise much influence on the pain or blindness. In ten minutes I gave her three more, and in the next ten minutes three more. In thirty minutes from the time of giving the first three seed, her vision was perfect and she had no pain. She was bitten at 5 P.M. She ate her supper at the table after dark. Next morning she was out at work, and has suffered no inconvenience since.

"I have reason to believe that they are a sure and ready antidote for the bite of all reptiles."

This plant, which my analysis makes the cnicus benedictus, or blessed thistle, is not a native of this continent, although it is naturalized in some localities, but is from Asia and Southern Europe. I may note the rather odd circumstance that all American writers and authors give it the name of centaurea benedicta, while it is known as above to all European authors. Dr. Porcher, in "Resources of the Southern Fields and Forests," states that it is naturalized in the neighborhood of Charleston, South Carolina, and says that the leaves are used in intermittent fever. A similar use of the plant is suggested in the "United States Dispensatory" of Wood & Bache.

Dr. J. G. Westmoreland, editor of this JOURNAL, in his work, "Acology and Therapeutics," introduces the blessed thistle in the character of a remedy for epilepsy. He believes it to act as a brain tonic, and says that it was tested during our late war by Dr. W. F. Westmoreland, and found by him to be valuable. This being true, why should it not serve a good purpose in other neurotic troubles, especially in such as have a convulsive tendency? In the "Acology and Therapeutics," the following way of using it is recommended in epilepsy:

"Boil sixty grains of the dried leaf in one pint of water for a few moments. Of this decoction, give three or four ounces three times a day."

The reader will find that the blessed thistle is mentioned by many medical writers, and he is referred to them for further information. But no mention is anywhere made of it as a remedy for the bite of poisonous serpents. I propose to plant some of the seed next spring, if all goes well with me, and will then, if I can, test its virtues by experiment. In the meantime, if any one desires to try its value, I presume that a few seed can be obtained of Dr. J. A. Shaffer, of Gainesville, Georgia.

In the course of my botanical studies, I have found a number of plants which, in their different localities, have a high reputation as remedies for snake bites, and as I am now engaged with this subject, I will record their names and leave them to the future experimenter.

The platanthera stricta is an orchid which grows abundantly near the sea-coast, but thins out up the country until it is wanting in the latitude of Atlanta. This plant by some authors is called habenaria cristata. I can only repeat what is told me of it. In Wayne and Glynn and other portions of lower Georgia, I have been very much interested to see how strong the faith was in this plant. Indeed, I was assured by them, so great was their faith, that the rattlesnake would not bite a man if he had the root in his pocket. One gentleman, a great deer hunter, told me that he had cured his dogs with it when bitten. The tincture of the root was used.

The baptisia pertoliata, one of the pea tribe of plants, and generally found growing in the same localities as the platanthera, has a like reputation, but is not so well known. The baptisia grows on the high and dry sandy ridges, while the platanthera prefers lower and damp situations.

Prof. W. T. Feay, a superior botanist, in a letter to me some years since, tells of the use of the baptisia as follows: "A gentleman in Florida had a negro woman bitten by a rattlesnake. A physician was immediately sent for. But a neighbor in the mean time recommended the use of this plant in decoction. It was done, and when the physician came the negro was well."

The amsonia ciliata, which I have seen only in Montgomery and adjoining counties in Georgia, is another of these remedies. Also the lycopus virginicus, a small plant, which I think may be found on nearly every stream in Georgia.

The following plants are mentioned in works on pure botany, as possessing antidotal virtues in the rattlesnake's bite: the liatris squarrosa, the nabalus albus, and the agave virginica.

The following quotation is taken from a little book bearing the title, "Life Among the Indians," and written by J. B. Finley, who was a missionary to the tribes which occupied southern and eastern Ohio, before its settlement by the whites: "We had not proceeded more than four miles till he was bit by a rattlesnake between the heel and ankle, his leggin not being tied down to his moccasin. He immediately killed the snake, and then went a few steps and pulled up a weed resembling a flax stalk, only not so tall. He took the root and chewed and swallowed some of it. The rest he applied to the wound. In a few minutes he became very sick, and began to vomit, and threw up something green and stringy, like poison. He then made the second application, and the third; and in an hour went on his journey without any difficulty. The bite did not swell more than if he had been stung by a wasp or bee. This herb has a yellow root, about the thickness of a darning needle. The stalk is single, about nine inches long, and its leaves resemble those of the flax stalk."

It seems to me that this plant could be easily identified from this description, by any botanist, and I would suggest that it be sought for on or near the Muskingum river, in Ohio. At any rate it will be found, if Mr. Finley can be relied on, somewhere in the lower part of that State. I cannot now give more particulars, as I made the above extract from the book while reading it about a year since, and am not now able to find the book, although I am very sure that I then got it from the Young Men's Library in Atlanta. But it cannot be found now. Some member of the Muskingum Medical Society, whether a botanist or not, could perhaps ascertain something about the plant. Would Dr. L. C. McElroy, or some other member, investigate the matter and publish the result?

I am satisfied that the blessed thistle will serve every purpose, but there can be no harm in multiplying reliable medicines.

I now come to the most difficult part of my work, namely, to offer a plausable explanation of the *modus operandi* of these remedies when they relieve a patient of the terrible suffering and death which result from the bite of venomous serpents. When a man is bitten, the poison which is injected into the wound through the fangs of the serpent is very quickly absorbed and distributed by the arteries, through the whole system, to wither and destroy the vitality of every part. I am disposed to believe that these remedies, when they do answer the purpose at all, do it on the antidotal principle. Wherever in the system they meet the poison, they at once neutralize it, as fully and as effectually as an alkali will neutralize an acid. It is very evident to me, that these poisons destroy life by their direct action upon the vital nerve centres, by either paralyzing or destroying their function. I must think that these nerve centres escape organic injury almost wholly, and are only injured functionally; for if it were otherwise, it would be very certain that no person who is bitten by a poisonous snake could, by any possibility, recover from it for some time, and not until the organic lesion of the centres had been healed. And yet it is quite a common case, as in the one above mentioned by Dr. Shaffer, for the patient to recover perfectly within a few hours. How could this possibly be if the nerve centres suffered an organic lesion?

I think that the symptoms in a bitten person all point to destroyed or perverted function of the nerve centres, as the principal effect of the poison. If this opinion be true, then it seems to me that we cannot well reach any other conclusion than that the remedy antidotes the poison by chemically neutralizing it. Let us take the only other possible view and see how it answers. The only other way by which a remedy can counteract a poison in these cases, is by leaving the poison itself alone, and relieving or remedying the evil it does. In its passage through the system, the poison comes upon a nerve centre and straightway throttles it as we may say. But the remedy in quick pursuit, finds the centre disabled, and immediately, by its own inherent virtue, restores it to health. And in this manner the remedy pursues the poison, undoing the evil it has done, until finally the poison is either exhausted, or, what is more probable, it is expelled from the system through some of the emunctories. Is this a reasonable view of the action of the poison and its remedy? I cannot think that any one will attempt to maintain it.

I must say that it is highly probable that alcohol and ammonia, in ther earlier effects in these cases, do act by directly sustaining the nerve centres until the first danger is passed. But the fact that neither the alcohol itself, nor any of its secondary products, can be detected in the body afterwards, would seem to point to the fact that it has entirely disappeared in neutralizing the poison. And this could scarcely be true, unless there was some chemical operation taking place.

